# Astragaloside IV Blunts Epithelial–Mesenchymal Transition and G2/M Arrest to Alleviate Renal Fibrosis via Regulating ALDH2-Mediated Autophagy

**DOI:** 10.3390/cells12131777

**Published:** 2023-07-04

**Authors:** Dong Li, Yuzhe Liu, Quancao Zhan, Yan Zeng, Ze Peng, Qifeng He, Qi Tan, Wenfu Cao, Shang Wang, Jianwei Wang

**Affiliations:** 1 Chongqing Key Laboratory of Traditional Chinese Medicine for Prevention and Cure of Metabolic Diseases, College of Traditional Chinese Medicine, Chongqing Medical University, Chongqing 400016, China; 2 College of Basic Medical Sciences, Chongqing Medical University, Chongqing 400016, China

**Keywords:** renal fibrosis, astragaloside IV, ALDH2, autophagy, AKT/mTOR

## Abstract

Previous studies show that astragaloside IV (ASIV) has anti-renal fibrosis effects. However, its mechanism remains elusive. In this study, we investigated the anti-fibrosis mechanisms of ASIV on chronic kidney disease (CKD) in vivo and in vitro. A CKD model was induced in rats with adenine (200 mg/kg/d, i.g.), and an in vitro renal fibrosis model was induced in human kidney-2 (HK-2) cells treated with TGF-β1. We revealed that ASIV significantly alleviated renal fibrosis by suppressing the expressions of epithelial–mesenchymal transition (EMT)-related proteins, including fibronectin, vimentin, and alpha-smooth muscle actin (α-SMA), and G2/M arrest-related proteins, including phosphorylated p53 (p-p53), p21, phosphorylated histone H3 (p-H3), and Ki67 in both of the in vivo and in vitro models. Transcriptomic analysis and subsequent validation showed that ASIV rescued ALDH2 expression and inhibited AKT/mTOR-mediated autophagy. Furthermore, in ALDH2-knockdown HK-2 cells, ASIV failed to inhibit AKT/mTOR-mediated autophagy and could not blunt EMT and G2/M arrest. In addition, we further demonstrated that rapamycin, an autophagy inducer, reversed the treatment of ASIV by promoting autophagy in TGF-β1-treated HK-2 cells. A dual-luciferase report assay indicated that ASIV enhanced the transcriptional activity of the ALDH2 promoter. In addition, a further molecular docking analysis showed the potential interaction of ALDH2 and ASIV. Collectively, our data indicate that ALDH2-mediated autophagy may be a novel target in treating renal fibrosis in CKD models, and ASIV may be an effective targeted drug for ALDH2, which illuminate a new insight into the treatment of renal fibrosis and provide new evidence of pharmacology to elucidate the anti-fibrosis mechanism of ASIV in treating renal fibrosis.

## 1. Introduction

Chronic kidney disease (CKD) is a progressive disease characterized by abnormalities of the structure and function of the kidney [1,2,3], caused by a number of illnesses, including diabetes mellitus, interstitial nephritis, glomerulonephritis, obstructive nephropathy, and polycystic kidney disease [4]. With incidences rising yearly, CKD is quickly becoming one of the serious illnesses endangering human health worldwide, affecting 26 to 30 million individuals [5,6]. With the progression of CKD into end-stage renal disease (ESRD), renal replacement therapies, such as dialysis and transplantation, are usually applied for patients. Healthcare costs for CKD patients, however, are more than twice as expensive as those for ESRD (USD 49 billion vs. USD 23 billion). Additionally, CKD is also related to accelerated aging and all-cause and cardiovascular mortality, which may further increase the burden on healthcare [7,8].

Renal fibrosis, the ultimate histopathologic manifestation of CKD, is strongly associated with the deterioration of renal function and the long-term prognosis in CKD patients [9,10,11]. It has been reported that renal fibrosis is characterized by epithelial–mesenchymal transition (EMT), the most important extracellular matrix-related process, as well as G2/M arrest [12,13]. In addition, it has been demonstrated that crosstalk exists between EMT and the G2/M cycle in the process of renal fibrosis [14]. However, extremely few and inadequate therapies are available to slow CKD progression and prevent CKD-related renal fibrosis [15]. Hence, studies aiming at therapeutic targets and experimental drugs for CKD are desperately needed.

Autophagy is a highly conserved cellular degradation mechanism, destroying and removing damaged organelles, cytoplasm, and even pathogens [16] and is responsible for maintaining cellular homeostasis and cell survival [17,18]. However, autophagy may be a double-edged sword in renal disease processes [19,20,21]. The results that autophagy leads to may vary depending on the phase and severity of the renal injury. For instance, moderate autophagic activity might maintain cellular homeostasis in early injured kidneys, whereas excessive or high autophagic activity may stimulate renal cell senescence and promote renal fibrosis by increasing the secretion of profibrotic cytokines in severely damaged kidneys [22,23]. In addition, it has been reported that in vivo and in vitro late treatment with the autophagy inhibitor 3-methyladenine (3-MA) significantly inhibits EMT and reduces the number of tubular cells arrested at the G2/M phase in the CKD model, which highlights the therapeutic potential of autophagy in renal fibrosis [24].

Acetaldehyde dehydrogenase 2 (ALDH2) is a key enzyme for mitochondria to maintain normal function [25]. It has been shown that ALDH2 has protective effects on diseases such as heart failure, atherosclerosis, and other ailments by reducing oxidative stress, inhibiting inflammation, promoting autophagy, and decreasing apoptosis [26]. The activation of ALDH2 can suppress high-glucose-induced fibrosis and necroptosis in primary rat cardiomyocytes [27]. Opposingly, ALDH2 deficiency exacerbates cardiac fibrosis by promoting the mobilization and homing of bone marrow fibroblast progenitor cells [28]. It was reported that ALDH2 knockout mice were accompanied by a deterioration in renal function and the apoptosis of the renal tubular epithelial cells (RTECs) in cisplatin-injection-induced acute kidney disease [29]. In addition, ALDH2 can also improve the disease process by regulating autophagy. ALDH2 protects against LPS-induced cardiac dysfunction by suppressing ER stress and autophagy [30]. ALDH2 may also regulate autophagy via the Akt-mTOR pathway to attenuate renal ischemia–reperfusion injury during hypothermic machine perfusion [31]. However, the role of ALDH2 in renal fibrosis of the CKD model is unclear.

Astragaloside IV (ASIV, 3-O-β-D-xylopyranosyl-6-O-β-D-glucopyranosyl cycloastragenol), a natural saponin extracted from *Astragali radix*, has been reported to have anti-inflammatory, anti-cancer, anti-oxidative, anti-fibrosis, and immune-regulatory functions [32]. It has been reported that in vivo ASIV can decrease renal mass loss, EMT, and the infiltration of inflammatory cells in UUO-induced renal fibrosis, and in vitro ASIV can significantly attenuate TGF-β1-induced fibrosis in human kidney-2 (HK-2) cells [33,34]. However, the underlying mechanism of ASIV in the treatment of renal fibrosis induced by adenine remains unclear. Herein, our new evidence suggests that it is by regulating ALDH2-mediated autophagy that ASIV mitigates EMT and G2/M arrest in renal fibrosis. Thus, ASIV might be a potential therapeutic candidate for adenine-induced renal fibrosis.

## 2. Materials and Methods

### 2.1. Reagents and Antibodies

Adenine (A8330) was obtained from Solarbio Life Science (Beijing, China). Recombinant human TGF-β1 protein (10804-HNAC) was purchased from SinoBiological (Beijing, China). Rapamycin (HY-10219) was obtained from MedChemExpress Biotech Co., Ltd. (Monmouth Junction, NJ, USA). Antibodies of fibronectin (ab268020), α-SMA (ab7817), ALDH2 (ab108306), and Ki67 (ab16667) were bought from Abcam (Cambridge, MA, USA). p-ULK1^ser757^ (14202s), p-AKT (4060s), SQSTM1 (5114s), and AKT (4691s) were from Cell Signaling Technology (Danvers, MA, USA). Vimentin (60330-1-Ig), p53 (60283-2-Ig), p-H3^ser10^ (66863-1-Ig), p-mTOR (67778-1-Ig), mTOR (66888-1-Ig), ULK1 (20986-1-AP), Lc3 (14600-1-AP), and GAPDH (10494-1-AP) were from Proteintech (Chicago, IL, USA). ATG7 (ET1610-53) and Beclin-1 (R1509-1) were from HuaBio (Hangzhou, China). E-cadherin (A20798) was from ABclone Technology (Wuhan, China). p-p53^ser15^ (530083) was from Zen-Bioscience (Chengdu, China). p21 (A5163) was brought from Bimake (Houston, TX, USA). Anti-α-tubulin (M03989-2) was from Boster (Wuhan, China). All other reagents and chemicals, unless indicated, were obtained from Sangon Biotech (Shanghai, China).

### 2.2. Drug Preparation

ASIV (purity ≥98.0%, CAS: 84687-43-4), purchased from Jingzhu Biotechnology (Nanjing, China), was dissolved in 0.5% sodium carboxymethyl cellulose (CMC-Na) solution for the in vivo experiments.

### 2.3. Animal Procedures

**Figure 1:**
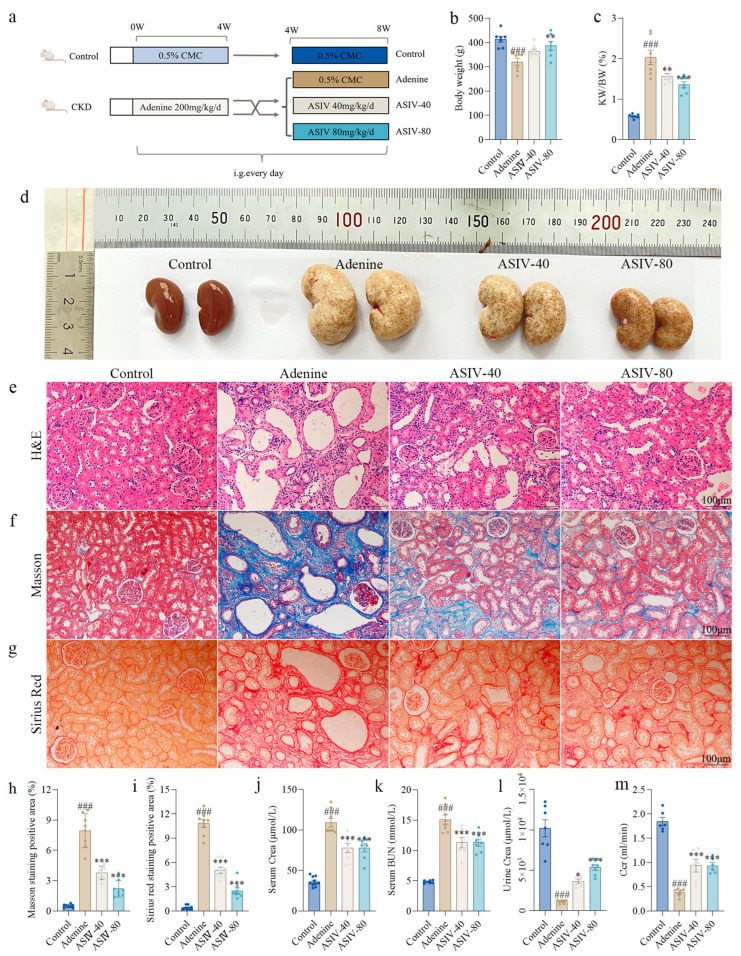
ASIV alleviates renal pathological damage and renal function in CKD rats. (**a**) Procedures for animal experiments. (**b**) Body weight and (**c**) kidney index (kidney weight/body weight) were measured when the rats were sacrificed. (**d**) The difference using macroscopic observation of kidneys in groups of the control, the adenine, the ASIV-40 (40 mg/kg/d), and the ASIV-80 (80 mg/kg/d) after sacrifice. (**e**) Hematoxylin and eosin (H&E) staining, (**f**) Masson staining, and (**g**) Sirius Red staining for renal tissues. (**h**) Masson trichrome-positive areas (%) in renal tissues. (**i**) Sirius Red-positive areas (%) in renal tissues. (**j**) Serum creatinine (Crea), (**k**) serum blood urea nitrogen (BUN), (**l**) urine Crea, and (**m**) creatinine clearance rate (Ccr) were determined when the rats were sacrificed. Data are presented as mean ± SEM (100 µm scale bar). One-way ANOVA was followed by Dunnett’s multiple comparisons test. ### *p* < 0.001 vs. the control group, * *p* < 0.05, ** *p* < 0.01, *** *p* < 0.001 vs. the adenine group.

Eight-week-old male SD rats (weighing 180 to 200 g) were purchased from the Laboratory Animal Center of Chongqing Medical University. The rats were housed under 12 h light/dark conditions at 22 ± 2 °C with free access to food and water. After an acclimatization of one week, the rat CKD models were established as described in previous research [35]. Specifically, the CKD group rats (*n* = 30) were administrated with adenine (200 mg/kg) for 4 weeks, and the control group rats (*n* = 10) were treated with equal volume of 0.5% CMC-Na solution. After 4 weeks of modeling, the CKD groups rats were randomly divided into three groups as follows: the adenine group (0.5% CMC-Na solution, *n* = 10); the ASIV-40 group (40 mg/kg ASIV, *n* = 10); and the ASIV-80 group (80 mg/kg ASIV, *n* = 10). Briefly, the animal procedures are shown in Figure 1a. After the rats were administrated with ASIV for 4 weeks, the rats were sacrificed, and their kidneys were harvested and stored at −80 °C. The body weight of rats was recorded every three days during animal experiments. All animal experiments were approved by the Ethics Committee of Chongqing Medical University (Chongqing, China), and the approval code is 2021069.

### 2.4. Biochemical Measurements

Four weeks after the administration of ASIV, 24 h urine of rats was collected using individual metabolic cages. The rats were anesthetized with 1% pentobarbital sodium after 12 h fasting with free access to water. Sera were obtained by centrifuging the blood at 3000 rpm for 20 min after coagulation. The serum creatinine, urea creatinine, and serum BUN were determined using commercially available assay kits (Nanjing Jiancheng Bioengineering Institute, Nanjing, China). All the determinations were carried out according to the instruction of manufacturers. Creatinine clearance rate (Ccr) was calculated using the following formula: urine creatinine (Ucr) (mg/dL) × urine volume per minute (mL/min)/serum creatinine (Scr) (mg/dL).

### 2.5. Hematoxylin and Eosin (H&E) Staining

The rat kidneys were photographed and fixed in 4% paraformaldehyde. Then, tissues were dehydrated, embedded in paraffin blocks, cut into 5 μm sections, and mounted on slides. The sections were deparaffinized, rehydrated, washed with H_2_O, and stained using Hematoxylin–Eosin (H&E) Stain Kit (G1120, Solarbio, Beijing, China), according to the manufacturer’s instruction. Then, the slides were observed under a light microscope (Leica, Wetzlar, Germany).

### 2.6. Masson Staining and Sirius Red Staining

Masson’s trichrome staining (G1340, Solarbio, Beijing, China) and Sirius Red staining (G1472, Solarbio, Beijing, China) were carried out according to the manufacturer’s instructions, respectively. Then, the slides were observed under a light microscope (Leica).

### 2.7. Immunohistochemistry

Kidney tissues were fixed in 4% paraformaldehyde, embedded in paraffin, and sectioned (5 μm) onto slides. Immunohistochemistry was carried out using following routine procedures with primary antibodies against ALDH2 (ab108306, Abcam, 1:500). Negative controls were treated identically but without primary antibody. The sections were washed with PBS and incubated with goat anti-rat immunoglobin G-horseradish peroxidase (IgG-HRP). After diaminobenzidine (DAB) staining (ZLI-9017, ZSGB-BIO, Beijing, China), images were captured under a light microscope (Leica).

### 2.8. Immunofluorescence

Briefly, for kidney tissues, 8 μm thickness cryosections or HK-2 cells were fixed with 4% paraformaldehyde for 30 min at room temperature, permeabilized with 0.1% Triton X-100 for 10 min. The slides were blocked with 10 % goat serum for 1 h at room temperature, followed by incubation with primary antibody involving rabbit monoclonal to fibronectin (1:500), mouse monoclonal to α-SMA (1:1000), mouse monoclonal to p-H3^ser10^ (1:100), Ki67 (1:200), and rabbit polyclonal to Lc3 (1:500) in a humidified dark box at 4 °C overnight. After incubation with secondary antibody (Abbkine, Wuhan, China) for 1 h at room temperature, cell nucleus was stained with 4, 6 diamidino-2-phenylindole (DAPI) for 10 min and then was observed under a fluorescence microscope (Leica).

### 2.9. RNA-Seq Analysis

Total RNA was extracted from rat kidney samples (each group containing 3 samples from the control, adenine, and ASIV-80 groups), according to the manufacturer’s instruction. After qualification and quantification, mRNA was purified using oligo(dT)-attached magnetic beads and then was fragmented into small pieces in corresponding buffer. Subsequently, the cDNA was synthesized using random hexamer-primed reverse transcription. The quality control of product was performed on the Agilent Technologies 2100 bioanalyzer. The products from previous step were heated, denatured, and circularized using the splint oligo sequence to obtain the final library. Finally, the library was sequenced on BGIseq500 platform (BGI-Shenzhen, China) and yielded paired-end 50 bases reads. The sequencing data were filtered using SOAPnuke (v1.5.2) to obtain clean reads, which were stored in FASTQ format. The clean reads were mapped to the reference genome and aligned to the reference coding gene set, and then, expression level of gene was calculated using RSEM (v1.2.12). The differentially expressed gene (DEG) analysis was performed with (1) a threshold of fold change ≥1.5 and (2) a Q-value ≤ 0.05. Then, the DEGs were analyzed using gene clustering analysis and gene set enrichment analysis (GSEA).

### 2.10. RNA Extraction and RT-PCR

To extract total RNA, the rat renal tissues (50 mg) or cultured HK-2 cells were homogenized in 500 μL TRIzol^®^ reagent (Invitrogen, Waltham, MA, USA). The total RNA pellet was dissolved in diethylpyrocarbonate (DEPC) water. The quality and quantity of total RNA were detected using ultraviolet absorption (optical density, 260 nm/280 nm) with a NanoDrop 2000 (Thermo Scientific, Waltham, MA, USA). An equal amount of total RNA (500 ng) was reverse transcribed to cDNA using Evo M-MLV RT Master Mix (Accurate Biotechnology, Hunan, China). Quantitative real-time PCR (qRT-PCR) was performed on Bio-Rad CFX 96 (Bio-Rad, Hercules, CA, USA) with SYBR^®^ Green Pro Taq HS Premix II (Accurate Biotechnology). The relative mRNA expression levels were assessed using the 2^−ΔΔCq^ method and normalized using gene GAPDH (used as an internal control gene). The primers were designed from IDT DNA Technology (Coralville, IA, USA) and synthesized by Sangon Biotech, Co., Ltd. (Shanghai, China). The primer sequences used for PCR are shown in Table S1.

### 2.11. Western Blotting

Total proteins from HK-2 cells or renal tissues were obtained in cold RIPA lysis buffer (Beyotime, Haimen, China) containing 1 mmol/L phenyl methane sulfonyl fluoride (PMSF, Beyotime, Haimen, China), 1% protease inhibitor cocktail, and 1% phosphatase inhibitor cocktail (Biotool, Houston, TX, USA) and centrifuged at 12,000× *g* for 15 min at 4 °C to remove debris. Protein concentrations were estimated using the bicinchoninic acid (BCA) protein assay kit (Beyotime, Haimen, China), and the protein extracts were heat-denatured in SDS-PAGE sample loading buffer (Beyotime, Haimen, China). Equal amounts of protein (40 μg/lane) from each sample were separated on 8–15% Tris-Glycine SDS-Page. The proteins were subsequently transferred onto a 0.22 μm polyvinylidene fluoride (PVDF) membrane (Merck Millipore, Darmstadt, Germany). After blocking with 5% non-fat dry milk for 1 h, the membrane was incubated with the primary antibodies overnight at 4 °C, followed by incubation with respective indicated secondary antibodies at room temperature for 1 h. The protein bands were detected using enhanced chemiluminescence (ECL) reagent (Millipore, Burlington, MA, USA), and the ratio of proteins was calculated using densitometry with ImageJ software 1.53a for Mac OS (Bethesda, MD, USA). Band densities were normalized using the expression level of glyceraldehyde 3-phosphate dehydrogenase (GAPDH).

### 2.12. Cell Culture and Treatment

The human kidney-2 (HK-2) cells line were obtained from the cell bank of the Chinese Academy of Sciences (Shanghai, China), originally from the American Type Culture Collection (ATCC, Manassas, VA, USA). The HK-2 cells were cultured in Dulbecco’s modified Eagle’s media (DMEM)/F12 (Gibco, Billings, MT, USA) containing 10% fetal bovine serum (FBS, Gibco, USA), and 1% penicillin and streptomycin (Gibco, USA) and were under standard cell culture conditions of 5% CO2 and 95% humidity at 37 °C. For in vitro CKD model, HK-2 cells were stimulated with TGF-β1 (10 ng/mL) for 48 h [36]. Rapamycin, an mTOR inhibitor, was used to induce in vitro autophagy as previously described [37]. Untreated HK-2 cells were used as controls.

### 2.13. Cell Viability Determination

HK-2 cells were seeded in 96-well plates (5 × 10^3^ cells/well). Then, cells were treated with TGF-β1 (10 ng/mL) or TGF- β1 together with different concentrations of ASIV (10, 50, 100, 150 μM) for 48 h. Cell viability was measured using Cell Counting Kit-8 (CCK-8, Dojindo, Kumamoto, Japan), according to the manufacturer’s protocol. The absorbance at 450 nm was detected using a microplate reader.

### 2.14. siRNA Transfectiony

The sequence of ALDH2 siRNA was 5′-CAG AUC AUU CCG UGG AAU UTT-3′. Briefly, HK-2 cells were seeded into 6-well plates and transfected transiently with ALDH2 siRNA or control siRNA (Sangon Biotech, Shanghai, China) using Lipofectamine 2000 (Invitrogen, Waltham, MA, USA) according to the manufacturer’s instruction. After transfection, cells were then treated with TGF-β1 (10 ng/mL) or TGF-β1 together with ASIV (150 μM) for another 48 h. Finally, cells were harvested for following investigations.

### 2.15. Dual Luciferase Reporter Assay

HEK-293T cells were transfected using the ALDH2 promoter luciferase reporter vector (RiboBio, Guangzhou, China) using Lipofectamine 2000 transfection reagent according to the manufacturer’s instruction. Transfection with pRL Renilla luciferase reporter vector was used for normalization and to assess transfection efficiency. After transfection, cells were treated with ASIV (0, 50, 100, 150 μM) for 48 h and then were lysed using passive lysis buffer. In addition, the luciferase activities were separately measured using a fluorescence spectrophotometer (Infinite M200, Tecan, Männedorf, Switzerland) following the manufacturer’s instructions for the Dual-Luciferase Reporter Assay kit (Yeasen Biotechnology, Shanghai, China).

### 2.16. Molecular Docking Analysis

The crystal structure of ALDH2 was downloaded from the Protein Data Bank (RCSB, ID: 3INL). The structure of ASIV was downloaded from PubChem (CID: 13943297) in structure data file (SDF) format and converted into PDBQT format using ADFR suite 1.0. The binding site analysis of ALDH2 and ASIV was performed using Autodock Vina 1.12. The exhaustiveness of the search was set as 32, and other parameters were set as default. The pose with the highest score was visualized using PyMOL 2.3.

### 2.17. Statistical Analysis

Data were analyzed using GraphPad Prism 7.0. All values are presented as mean ± standard error of means (SEM) unless otherwise indicated. Comparisons among two groups were analyzed using Student’s *t*-test. Comparisons among groups (more than three groups) were analyzed using one-way ANOVA followed by Dunnett’s test. Cell experiments were repeated at least three times to ensure confidence in the results. *p* < 0.05 was considered statistically significant.

## 3. Results

### 3.1. ASIV Alleviated Renal Pathological Changes and Improved Renal Function in Adenine-Induced CKD Rats

Male SD rats were exposed to adenine to induce the CKD model. After 4 weeks of modeling, compared to the control group rats, a significant body weight loss was shown in the CKD group rats (Figure S1a). Serum creatinine and blood urea nitrogen (BUN) were determined to evaluate the renal function of the rats. Compared with the control group rats, both serum creatinine and BUN were elevated significantly in the CKD group rats (Figure S1b,c), indicating the deterioration of renal function. After 4 weeks’ treatment of ASIV, as shown in Figure 1b–d, compared with the rats in the control group, the adenine group rats had a significant body weight loss and a remarkable kidney index increase, accompanied by a significantly enlarged renal volume, a white and uneven surface, and fine granular appearance. Moreover, H&E staining showed that the adenine group rats had injury renal tubular, accompanied by shedding of the renal tubular epithelial cells, tubular atrophy, and dilatation of the tubular lumen (Figure 1e). Both Masson trichrome and Sirius Red staining were used to determine collagen fibers. The proportion of Masson trichrome staining-positive areas in the ASIV group (3.77% in the ASIV-40 group and 2.20% in the ASIV-80 group) were significantly lower than that of the adenine group (7.94%) (Figure 1f,h). Sirius Red staining revealed that the collagen-positive area decreased significantly after ASIV treatment (10.84% in the model group versus 5.02% in the ASIV-40 group and 2.50% in the ASIV-80 group) (Figure 1g,i). Additionally, the adenine-induced elevation of serum creatinine and BUN were significantly reversed by ASIV (Figure 1j,k). Additionally, compared with the control group rats, adenine led to significant decreases in urine creatinine and Ccr (Figure 1l,m). However, ASIV significantly recovered the urine creatinine and Ccr in CKD rats. Collectively, these results suggest that ASIV ameliorates renal pathological changes and renal function in adenine-induced CKD rats.

### 3.2. ASIV Alleviates EMT and G2/M Arrest in the Renal Tissues of CKD Rats

**Figure 2:**
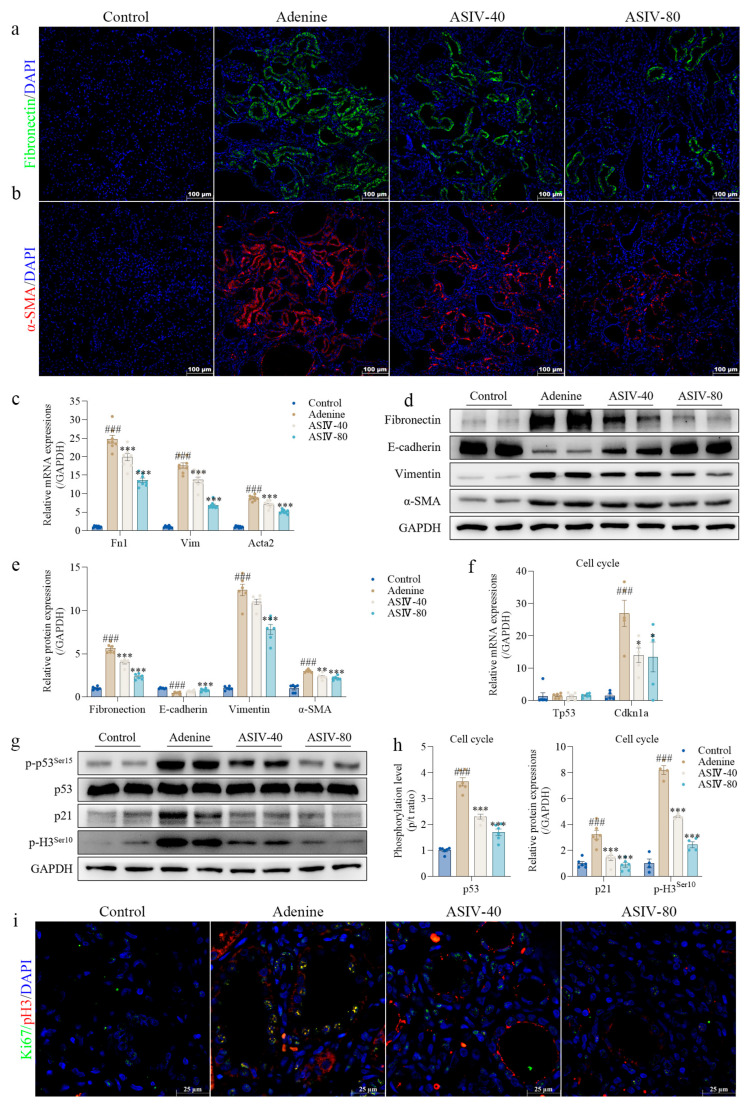
ASIV alleviates EMT and G2/M arrest in the renal tissues of CKD rats. Representative images of immunofluorescence staining for (**a**) fibronectin and (**b**) a-SMA in rat kidneys (*n* = 6, 100 µm scale bar). (**c**) Relative gene expression of Fn1, Vim, and Acta2 in the renal tissues of each group. (**d**) Protein expressions of fibronectin, E-cadherin, vimentin, and α-SMA in the renal tissues of each group using Western blotting. (**e**) Quantitative analysis of the relative protein expressions of fibronectin, E-cadherin, vimentin, and α-SMA in kidney tissues. (**f**) Relative gene expressions of Tp53 and Cdkn1a in renal tissues of each group using qRT-PCR. (**g**) Protein expressions of p-p53^ser15^, p53, p21, and p-H3^ser10^ in the renal tissues of each group using Western blotting. (**h**) Quantitative analysis of the phosphorylation level of p53 and the relative protein expressions of p21 and p-H3^ser10^ in the renal tissue. (**i**) Representative images of immunofluorescence double staining for Ki67 (green) and p-H3 (red) in the renal tissues of each group (*n* = 6, 25 µm scale bar). Data are presented as mean ± SEM. One-way ANOVA was followed by Dunnett’s multiple comparisons test. ### *p* < 0.001 vs. the control group, * *p* < 0.05, ** *p* < 0.01, *** *p* < 0.001 vs. the adenine group.

Previous evidence has demonstrated that renal fibrosis is closely related to EMT and G2/M arrest [38,39]. In the present study, compared with the control group rats, a significant increase in the mesenchymal cell markers, fibronectin, vimentin, and α-SMA and a remarkable decrease in the epithelial cell marker E-cadherin were found in the kidneys of the adenine group rats. However, ASIV significantly inhibited EMT by reversing the abnormal expressions above in the CKD rat kidneys (Figure 2c–e), which was further validated using immunofluorescence analysis for fibronectin and α-SMA in the rat kidneys (Figure 2a,b). In addition, we examined the expression of genes or proteins, including phospho-p53 (p-p53), p53, p21, and phospho-histone H3 (p-H3), which were strongly associated with G2/M arrest [40,41]. Compared with the control group rats, significant increases were found in the phosphorylation level of p53 and the expression of p21 and p-H3 in the kidneys of the adenine group rats. Similarly, ASIV also remitted G2/M arrest by inverting the expressions of the related genes and proteins above in the kidneys of the adenine-treated rats (Figure 2f–h). Additionally, we further detected the cell marker Ki67 for proliferation [42] and the cell marker p-H3 [43] for G2/M arrest using immunofluorescence. The results show that the expression of p-H3 and Ki67 both increased in the adenine group and were reversed by ASIV treatment (Figure 2i). Taken together, these data indicate that ASIV mitigates EMT and G2/M arrest in the kidney of CKD rats.

### 3.3. RNA-Seq Analysis Reveals the Mechanisms of ASIV on the Kidney in Adenine-Induced CKD Rats

**Figure 3:**
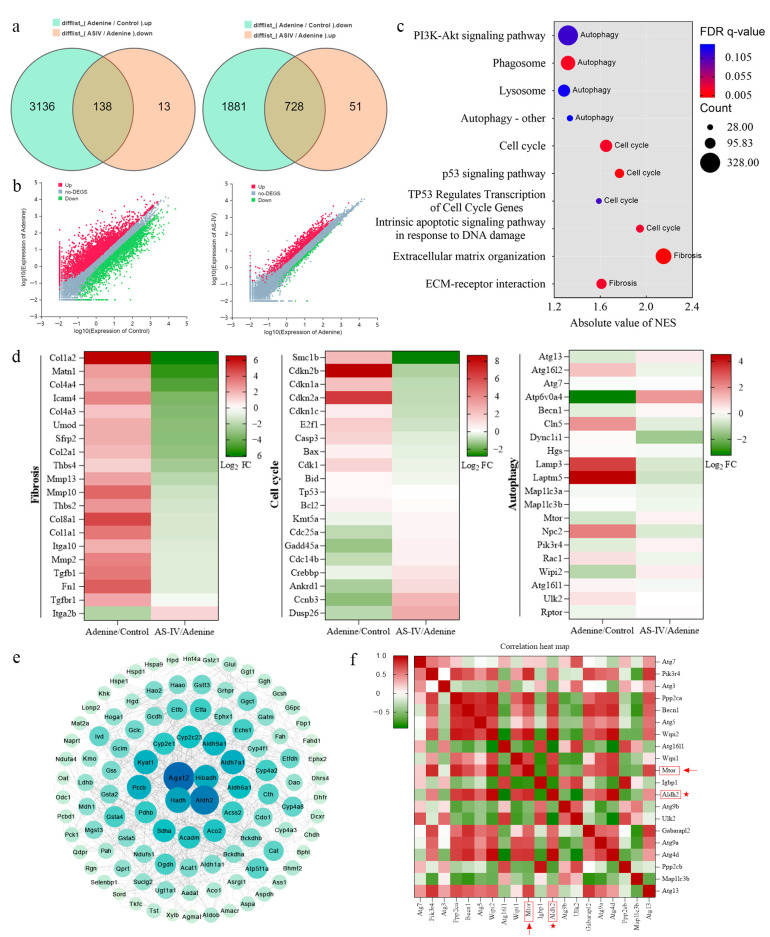
RNA-Seq analysis reveals the mechanisms of ASIV on the kidneys of adenine-induced CKD rats. (**a**) Venn diagram of the overlapped differentially expressed genes (DEGs) between the control group vs. the adenine group and the adenine group vs. the ASIV-80 group. (**b**) Scatter plot of the upregulated and downregulated DEGs in the control group vs. the adenine group and the adenine group vs. the ASIV-80 group. (**c**) Bubble histogram for the results of gene set enrichment analysis (GSEA). (**d**) Heat maps of gene expression profiles related to fibrosis, cell cycle, and autophagy based on the RNA-Seq data set. (**e**) Protein–protein interaction (PPI) network. (**f**) Heat map showing the relationship between ALDH2 and autophagy-related genes based on the RNA-Seq data set.

To systematically elucidate how ASIV treats renal fibrosis, we performed a transcriptomic analysis on the kidney tissues of the control group, the adenine group, and the ASIV-80 group rats. Compared with the control group, adenine treatment upregulated 3274 genes and downregulated 2609 genes, whereas ASIV treatment reversed 138 of the 3274 upregulated genes and 728 of the 2609 downregulated genes (Figure 3a,b). Gene set enrichment analysis (GSEA) revealed that ASIV-induced gene changes were mainly enriched in fibrosis, the cell cycle, and autophagy (Figure 3c,d). Moreover, a heat map based on the GSEA indicated that the renal genes involved in fibrosis, the cell cycle, and autophagy were significantly downregulated by ASIV in adenine-induced CKD rats (Figure 3d). To further determine the specific impact of ASIV on renal fibrosis from the molecular level, we integrated and analyzed the differentially expressed genes (DEGs) in RNA-Seq by performing a protein–protein interaction (PPI) network analysis using the STRING database (https://string-db.org, accessed on 12 February 2022). Specifically, the results show that four hub genes (Agxt2, ALDH2, Hadh, and Hibadh) were shown in the center of the PPI network (Figure 3e). After reviewing the literature, we found that the ALDH2 mRNA level was markedly downregulated in the kidneys of CKD patients compared with normal kidneys, while the other three genes were not reported [44,45]. In addition, it was reported that ALDH2 was associated with the regulation of autophagy [46]. Therefore, we made a heat map between ALDH2 gene and autophagy-related genes to show their relationship (Figure 3f). The depth of the color represents the correlation between the genes on the axes *X* and *Y*. Based on this concept, the heat map showed that the Aldh2 gene has a strong correlation with autophagy-related genes in the present study. Collectively, these results suggest that the remedial effects of ASIV on CKD rat kidneys might be involved in the alleviation of renal fibrosis, the regulation of ALDH2 expression, and the mediation of the cell cycle and autophagy.

### 3.4. ASIV Promotes ALDH2 Expression, Inhibits Autophagy, and Activates AKT/mTOR Signaling Pathway In Vivo

**Figure 4:**
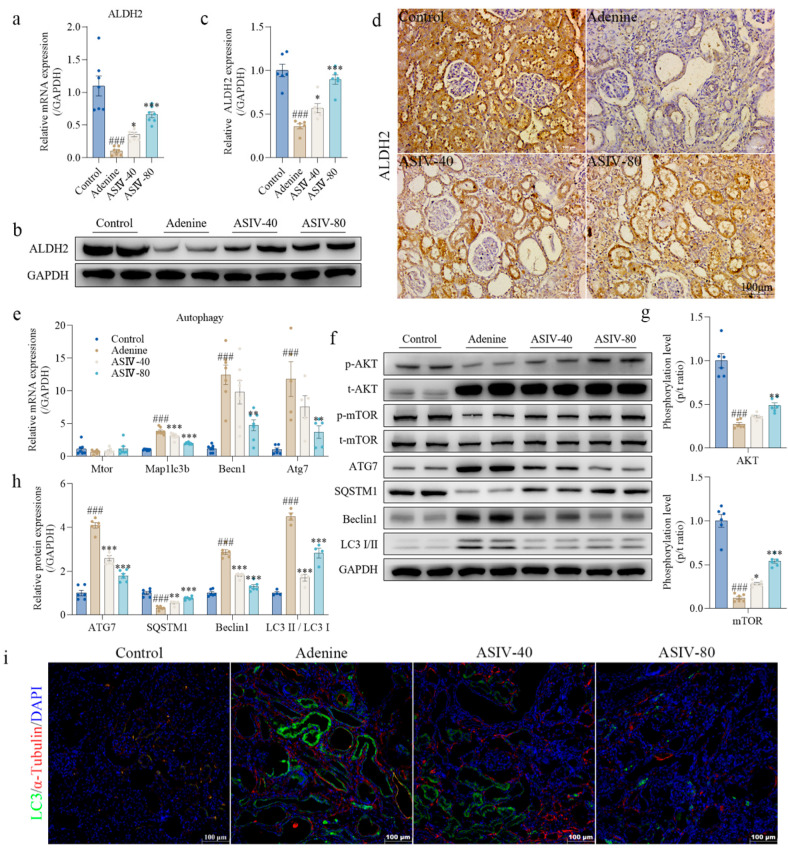
ASIV promotes ALDH2 expression, inhibits autophagy, and activates AKT/mTOR signaling pathway in vivo. (**a**) ALDH2 mRNA level in the renal tissues using qRT-PCR for each group. (**b**) Protein expression of ALDH2 in the renal tissues of each group using Western blotting. (**c**) Quantitative analysis of the relative protein expression of ALDH2 in the renal tissues. (**d**) Representative images of immunohistochemistry staining for ALDH2 in the rat kidneys (*n* = 6, 100 µm scale bar). (**e**) Relative gene expressions of Mtor, Map1lc3b, Becn1, and Atg7 in the renal tissues of each group (*n* = 6). (**f**) Protein expressions of p-AKT, t-AKT, p-mTOR, t-mTOR, ATG7, SQSTM1, Beclin1, and LC3 I/II in the renal tissues of each group using Western blotting. (**g**) Quantitative analysis of the phosphorylation levels of AKT and mTOR in the renal tissues. (**h**) Quantitative analysis of the relative protein expressions of ATG7, SQSTM1, Beclin1, and LC3 II/LC3 I in the renal tissues. (**i**) Representative images of immunofluorescence staining for LC3 I/II/α-tubulin in the rat kidneys (*n* = 6, 100 µm scale bar). Data are presented as mean ± SEM. One-way ANOVA was followed by Dunnett’s multiple comparisons test. ### *p* < 0.001 vs. the control group, * *p* < 0.05, ** *p* < 0.01, *** *p* < 0.001 vs. the adenine group.

To experimentally validate the transcriptomics results, we performed qPCR and Western blotting on the same cohort to determine the gene and protein expressions of ALDH2 in the kidney tissues of each group of rats. The results show that adenine decreased the mRNA and protein expression levels of ALDH2 in rat kidneys, which was significantly reversed by ASIV (Figure 4a–c), and this was further certified using immunohistochemistry analysis for ALDH2 in the renal fibrosis of rats (Figure 4d). Furthermore, to detect the autophagy level of rat kidneys, we used qRT-PCR and Western blotting to determine the gene or protein expressions of ATG7, SQSTM1, Beclin1, and LC3, which are usually used as indicators of autophagy [47]. The results show that adenine significantly increased the gene and protein expression levels of ATG7, Beclin1, and LC3, whereas ASIV significantly decreased their expression levels (Figure 4e,f,h). Compared with the control group, adenine significantly decreased the expression of SQSTM1 in the rat kidneys, which was significantly reversed by ASIV (Figure 4f,h). Similarly, the immunofluorescence analysis showed that compared with the control group, adenine significantly increased the expression of LC3 in the rat kidneys, which was significantly inhibited by ASIV (Figure 4i). Given that the AKT/mTOR signaling pathway is a key autophagy regulator in many pathophysiological processes associated with kidney disease [48,49], we used Western blotting to detect the activation of the AKT/mTOR pathway in the renal tissue, and the results show that the phosphorylation levels of AKT and mTOR were significantly decreased by adenine in the rat kidneys. However, ASIV significantly recovered the phosphorylation of AKT and mTOR (Figure 4f,g). Altogether, these data demonstrate that ASIV promotes the expression of ALDH2, inhibits autophagy, and activates AKT/mTOR signaling pathways in the kidneys of CKD rats.

### 3.5. ASIV Mitigates EMT and G2/M Arrest, Promotes ALDH2 Expression, Inhibits Autophagy, and Activates AKT/mTOR Signaling Pathway in TGF-β1-Stimulated HK-2 Cells

**Figure 5:**
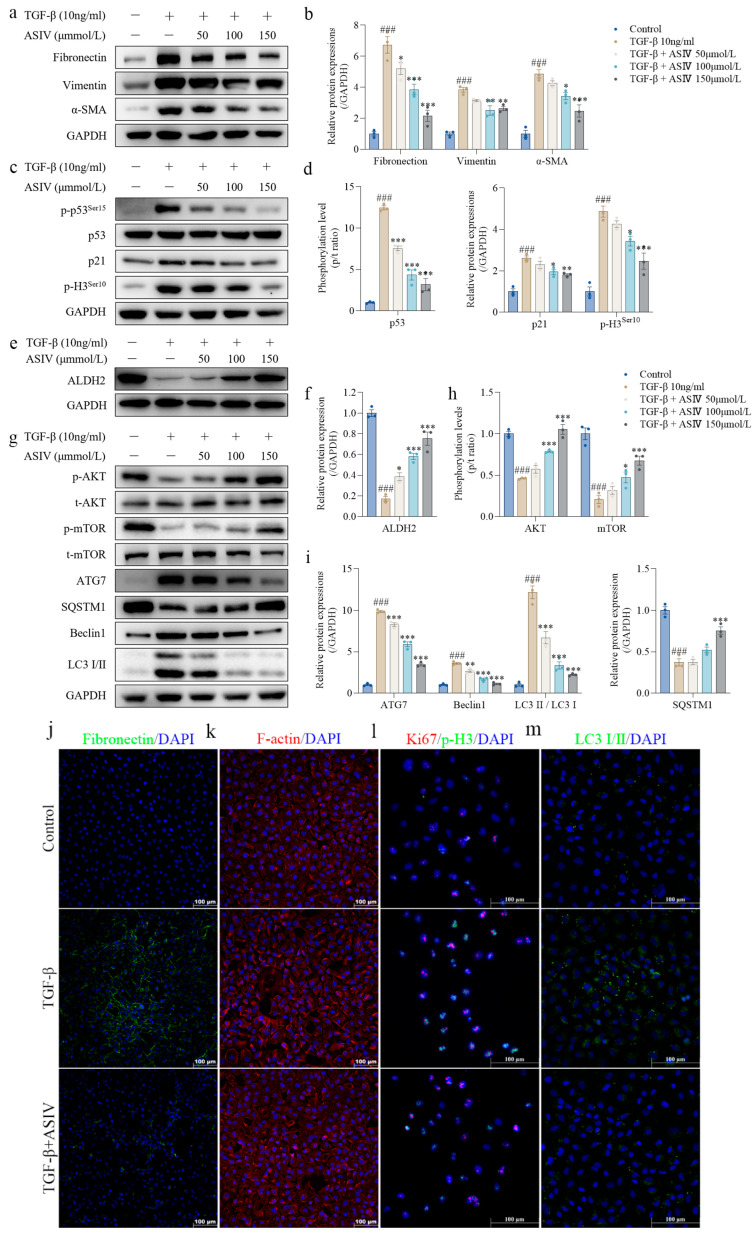
ASIV mitigates EMT and G2/M arrest, promotes ALDH2 expression, inhibits autophagy, and activates AKT/mTOR signaling pathway in TGF-β1-stimulated HK-2 cells. (**a**) Protein expressions of fibronectin, vimentin, and α-SMA in HK-2 cells using Western blotting. (**b**) Quantitative analysis of the relative protein expressions of fibronectin, vimentin, and α-SMA. (**c**) Protein expressions of p-p53^ser15^, p53, p21, and p-H3^ser10^ in HK-2 cells using Western blotting. (**d**) Quantitative analysis of the phosphorylation level of p53 and the relative protein expressions of p21 and p-H3^ser10^. (**e**) Protein expression of ALDH2 in HK-2 cells using Western blotting. (**f**) Quantitative analysis of the relative protein expression of ALDH2. (**g**) Protein expression of p-AKT, t-AKT, p-mTOR, t-mTOR, ATG7, SQSTM1, Beclin1, and LC3 I/II in HK-2 cells using Western blotting. (**h**) Quantitative analysis of the phosphorylation levels of AKT and mTOR in HK-2 cells. (**i**) Quantitative analysis of the relative protein expressions of ATG7, SQSTM1, Beclin1, and LC3 II/LC3 I in HK-2 cells. Representative images of immunofluorescence staining for (**j**) fibronectin, (**k**) F-actin, (**l**) Ki67 (red)/p-H3 (green), and (**m**) LC3 I/II in HK-2 cells. Data are presented as mean ± SEM (*n* = 3, 100 µm scale bar). One-way ANOVA was followed by Dunnett’s multiple comparisons test. ### *p* < 0.001 vs. the control group, * *p* < 0.05, ** *p* < 0.01, *** *p* < 0.001 vs. the TGF-β1 group.

To further investigate the underlying mechanism of ASIV on renal fibrosis, TGF-β1-stimulated HK-2 cells were used as a classical renal fibrosis model in vitro [50]. The CCK-8 results show that ASIV (10, 50, 100, and 150 μM) significantly improves cell viability in TGF-β1-stimulated HK-2 cells in a concentration-dependent manner (Figure S5a). Next, we evaluated the effect of ASIV on the phenotype of EMT and G2/M arrest in TGF-β1-stimulated HK-2 cells. Consistent with the in vivo results, TGF-β1 led to remarkable increases in the protein expressions of fibronectin, vimentin, and α-SMA in vitro, whereas ASIV significantly reversed these indicators of EMT (Figure 5a,b). In addition, the results show that TGF-β1 markedly increased the expression of p-p53, p21, and p-H3 in vitro, which were significantly reversed by ASIV (Figure 5c,d), indicating that ASIV effectively alleviated TGF-β1-induced G2/M arrest in vitro.

Then, we detected the ALDH2 protein expression in HK-2 cells, and the results show that TGF-β1 remarkably decreased the ALDH2 protein expression in HK-2 cells, whereas ASIV significantly increased ALDH2 protein expression in a concentration-dependent manner (Figure 5e,f). Furthermore, the phosphorylation levels of AKT and mTOR were markedly decreased in TGF-β1-treated HK-2 cells, accompanied by significantly upregulated expression levels of autophagy-related proteins, including ATG7, Beclin1, and LC3 II/LC3 I, and the downregulated expression level of SQSTM1(Figure 5g–i). However, ASIV significantly reversed the abnormal expressions of these autophagy-related indicator proteins and promoted the phosphorylation levels of AKT and mTOR in vitro (Figure 5g,h). Additionally, an autophagy flux blocker (chloroquine) was applied to verify the status of autophagy flux in the ASIV-induced treatment, and the results show that with the use of chloroquine, ASIV could also significantly inhibit autophagy in TGF-β1-stimulated HK-2 cells (Figure S5b,c).

Immunofluorescence staining was carried out to detect the effect of ASIV on the phenotype of EMT, G2/M arrest, and autophagy in TGF-β1-stimulated HK-2 cells. The results show that TGF-β1 leads to a significant increase in the expression of fibronectin (Figure 5j) and F-actin (Figure 5k) in HK-2 cells, which was significantly reversed by ASIV, indicating that ASIV mitigates TGF-β1-induced EMT in vitro. G2/M arrest affected the proliferation efficiency of the epithelial cells [51]. In the present study, to determine the cells in proliferation and/or arrested in G2/M phase, we used immunofluorescence double staining for ki67 and p-H3 to gauge the cell cycle distribution, and the results show that the expression of Ki67 and p-H3 increased in the TGF-β1-induced HK-2 cells and was reversed by ASIV treatment (Figure 5l). The immunofluorescence analysis for LC3 I/II showed that ASIV significantly decreased the expression of LC3 induced by TGF-β1 in vitro (Figure 5m). Taken together, these results further indicate that ASIV alleviates EMT and G2/M arrest, promotes the expression of ALDH2, inhibits autophagy, and activates the AKT/mTOR signaling pathway in TGF-β1-stimulated HK-2 cells.

### 3.6. ALDH2 siRNA Blocks the Beneficial Effects of ASIV on Renal Fibrosis In Vitro

**Figure 6:**
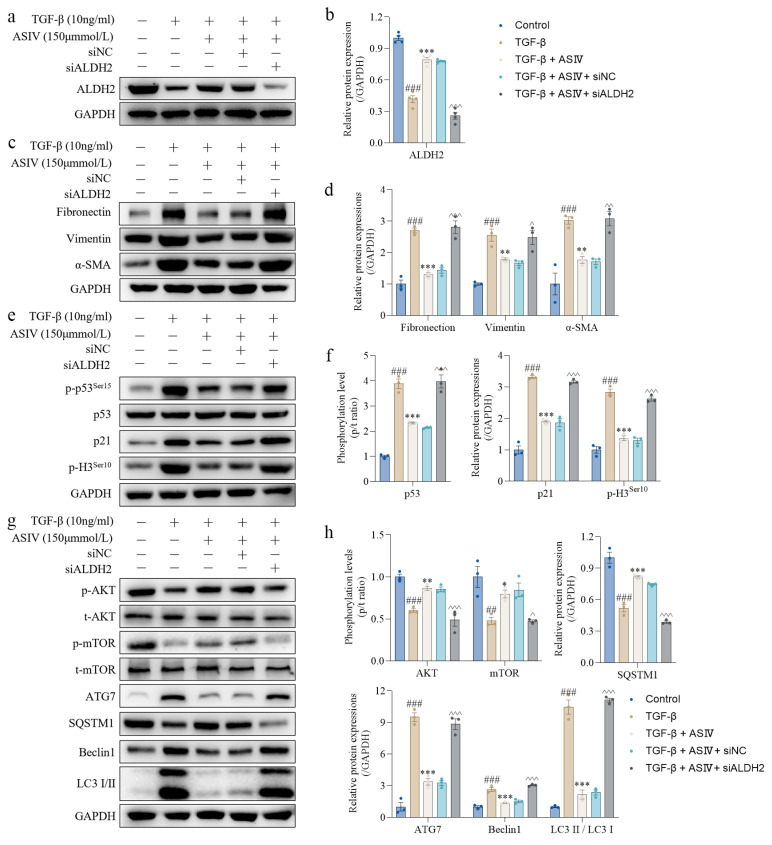

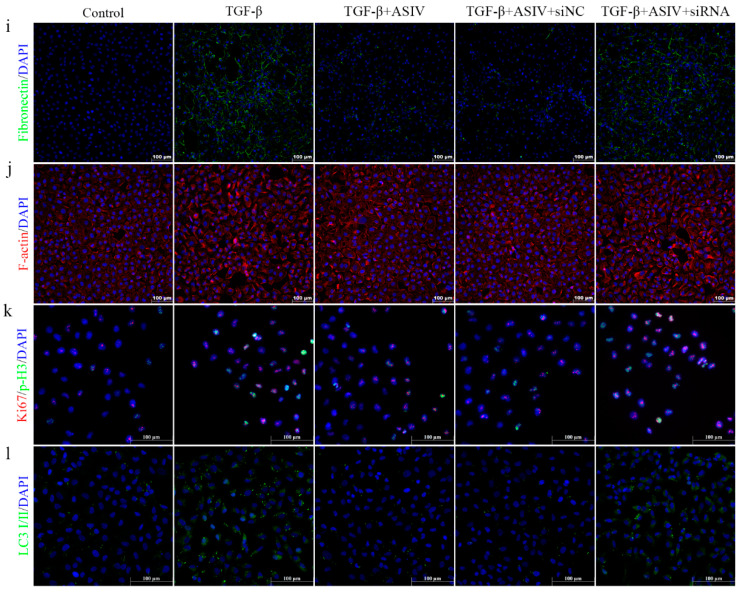
ALDH2 siRNA blocks the beneficial effects of ASIV on renal fibrosis in vitro. (**a**) Protein expression of ALDH2 in HK-2 cells using Western blotting. (**b**) Quantitative analysis of the relative protein expression of ALDH2. (**c**) Protein expression of fibronectin, vimentin, and α-SMA in HK-2 cells using Western blotting. (**d**) Quantitative analysis of the relative protein expressions of fibronectin, vimentin, and α-SMA. (**e**) Protein expression of p-p53^ser15^, p53, p21, and p-H3^ser10^ in HK-2 cells using Western blotting. (**f**) Quantitative analysis of the relative protein expressions of p-p53/t-p53, p21, and p-H3^ser10^ in HK-2 cells of each group using Western blotting. (**g**) Protein expression of p-AKT, t-AKT, p-mTOR, t-mTOR, ATG7, SQSTM1, Beclin1, and LC3 I/II in HK-2 cells of each group using Western blotting. (**h**) Quantitative analysis of the phosphorylation levels of AKT and mTOR and the relative protein expressions of ATG7, SQSTM1, Beclin1, and LC3 II/LC3 I. Representative image of immunofluorescence staining for (**i**) fibronectin, (**j**) F-actin, (**k**) Ki67 (red)/p-H3 (green), and (**l**) LC3 I/II in the HK-2 cells. Data are presented as mean ± SEM (*n* = 3, 100 µm scale bar). One-way ANOVA was followed by Dunnett’s multiple comparisons test. ## *p* < 0.01, ### *p* < 0.001 vs. the control group; * *p* < 0.05, ** *p* < 0.01, *** *p* < 0.001 vs. the TGF-β1 group; ^ *p* < 0.05, ^^ *p* < 0.01, ^^^ *p* < 0.001 vs. the TGF-β1 + ASIV group.

To further illustrate the role of the ALDH2 in ASIV treatment, small interference RNA was used to knock down ALDH2. The interference efficiency of ALDH2 siRNA is shown in Figure S6a,b. The results show that ALDH2 siRNA significantly restrained the ASIV-induced promoting effect on ALDH2 protein expression in TGF-β1-stimulated HK-2 cells, whereas the negative control siRNA did not (Figure 6a,b). Further results show that ALDH2 siRNA significantly offset the inhibiting effect of ASIV on the protein expressions of fibronectin, vimentin, and α-SMA in TGF-β1-stimulated HK-2 cells (Figure 6c,d). In addition, ALDH2 siRNA remarkably counteracted the suppressive effect of ASIV on the protein expressions of p-p53, p21, and p-H3 in vitro (Figure 6e,f). Furthermore, under the treatment of ALDH2 siRNA, ASIV not only failed to restrain the protein expressions of ATG7, Beclin1, and LC3 II/LC3 I but did not upregulate the phosphorylation levels of AKT and mTOR, as well as the protein expression of SQSTM1 in the TGF-β1-stimulated HK-2 cells (Figure 6g,h). Similarly, immunofluorescence staining showed that the remedial effects of ASIV on renal fibrosis in TGF-β1-stimulated HK-2 cells were all blocked by ALDH2 siRNA (Figure 6i–l). Altogether, these results suggest that ALDH2 is necessary for ASIV in mitigating EMT and G2/M arrest, inhibiting autophagy, and activating AKT/mTOR signaling pathways in vitro.

### 3.7. mTOR Signaling Pathway-Mediated Autophagy Is Required for the ASIV-Induced Alleviation of EMT and G2/M Arrest In Vitro

**Figure 7:**
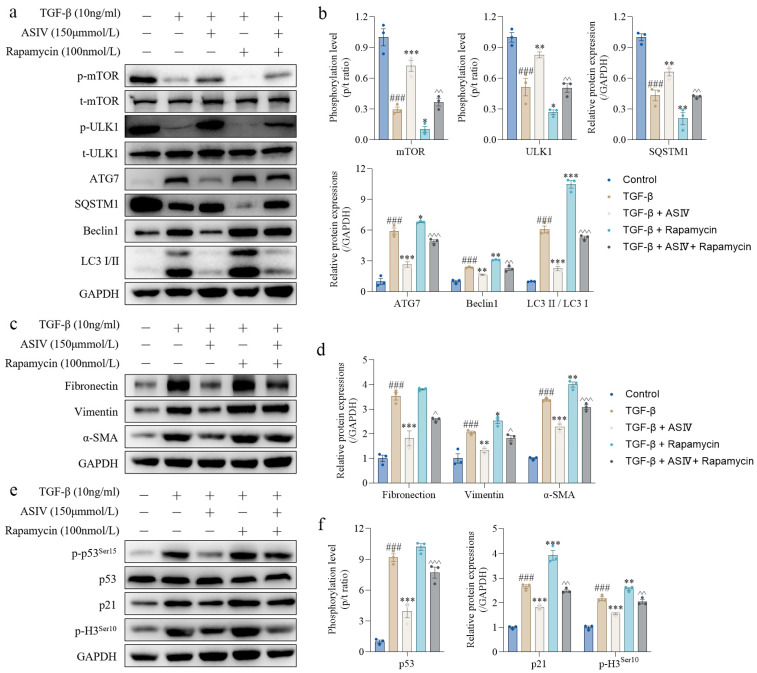

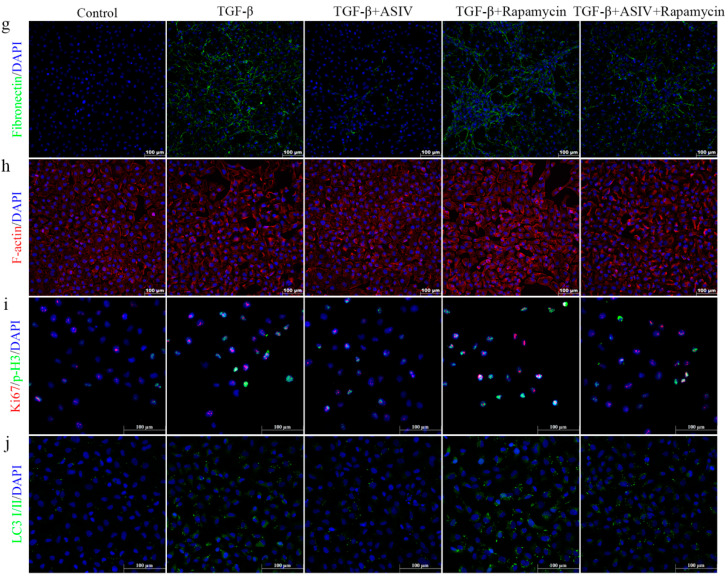
AKT/mTOR-signaling-pathway-mediated autophagy is required for the ASIV-induced alleviation of EMT and G2/M arrest in vitro. (**a**) Protein expressions of p-mTOR, t-mTOR, p-ULK1, t-ULK1, ATG7, SQSTM1, Beclin1, and LC3 I/II in HK-2 cells using Western blotting. (**b**) Quantitative analysis of the phosphorylation levels of mTOR and ULK1 and the relative protein expressions of ATG7, SQSTM1, Beclin1, and LC3 II/LC3 I. (**c**) Protein expressions of fibronectin, vimentin, and α-SMA in HK-2 cells using Western blotting. (**d**) Quantitative analysis of the relative protein expressions of fibronectin, vimentin, and α-SMA. (**e**) Protein expression of p-p53^ser15^, p53, p21, and p-H3^ser10^ in HK-2 cells using Western blotting. (**f**) Quantitative analysis of the phosphorylation level of p53 and the relative protein expressions of p21 and p-H3^ser10^. Representative images of immunofluorescence staining for (**g**) fibronectin, (**h**) F-actin, (**i**) Ki67 (red)/p-H3 (green), and (**j**) LC3 I/II in the HK-2 cells. Data are presented as mean ± SEM (*n* = 3, 100 µm scale bar). One-way ANOVA was followed by Dunnett’s multiple comparisons test. ### *p* < 0.001 vs. the control group; * *p* < 0.05, ** *p* < 0.01, *** *p* < 0.001 vs. the TGF-β1 group; ^ *p* < 0.05, ^^ *p* < 0.01, ^^^ *p* < 0.001 vs. the TGF-β1 + ASIV group.

Rapamycin, an mTOR inhibitor that can induce autophagy, was used to further investigate the role of mTOR-mediated autophagy in the anti-fibrotic effect of ASIV in vitro. The results show that the ASIV-induced phosphorylation of mTOR and ULK1, upregulation of the SQSTM1 protein, and downregulation of proteins, including ATG7, Beclin1, and LC3 II/LC3 I, were significantly inhibited by rapamycin in TGF-β1-stimulated HK-2 cells (Figure 7a,b). Then, we detected the effects of autophagy on the TGF-β1-induced phenotypes of EMT and G2/M arrest in vitro. The results show that rapamycin significantly blocked the restraining effect of ASIV on the protein expressions of fibronectin, vimentin, and α-SMA in vitro (Figure 7c,d). Additionally, under treatment with rapamycin, ASIV failed to decrease the protein expressions of p-p53, p21, and p-H3 in vitro (Figure 7e,f). Additionally, we observed the effect of rapamycin on the phenotype and investigated the effect of ASIV on the rapamycin-induced phenotype. The results show that rapamycin led to the phosphorylation of mTOR and the increasing expression of LC3 II/LC3 I, fibronectin, and p-H3 (Figure S7a,b), which indicated similar phenotypes induced by TGF-β1 in HK-2 cells. Furthermore, ASIV could significantly reverse these phenotypes induced by rapamycin. Similarly, immunofluorescence staining showed that rapamycin blunts the therapeutic effects of ASIV on renal fibrosis in TGF-β1-stimulated HK-2 cells (Figure 7g–j). Taken together, these data indicate that mTOR-mediated autophagy is necessary for the ASIV-induced mitigation of EMT and G2/M arrest in vitro.

### 3.8. ASIV Upregulates the Transcriptional Activity of ALDH2 Promoter, and Molecular Docking Predicts the Potential Interaction between ASIV and ALDH2 Protein

**Figure 8:**
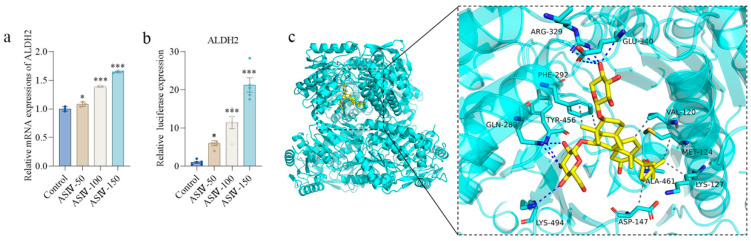
ASIV upregulates the transcriptional activity of ALDH2 promoter, and molecular docking predicts the potential interaction between ASIV and ALDH2. (**a**) Relative gene expression of ALDH2 in HK-2 cell using qRT-PCR. (**b**) Relative luciferase expression of ALDH2 promoter in HEK-293T cells. (**c**) Autodock Vina software predicts the ligand–protein interaction between ASIV and ALDH2. Data are presented as mean ± SEM. One-way ANOVA was followed by Dunnett’s multiple comparisons test. * *p* < 0.05, *** *p* < 0.001 vs. the control group.

We further determined the effects of ASIV on ALDH2 mRNA and the ALDH2 promoter in vitro. The results of qRT-PCR show that ASIV increased the ALDH2 mRNA level in HK-2 cells in a concentration-dependent manner (Figure 8a), and the results of dual-luciferase reporter assay show that ASIV significantly enhanced the transcriptional activity of the ALDH2 promoter in a concentration-dependent manner (Figure 8b), which indicated indirect regulation on a transcriptional level.

In addition, we further performed molecular docking between ASIV and the ALDH2 protein. The docking calculation result, with the side chains of the residues around the binding pocket set as rigid, shows that ASIV exhibited suitable steric complementarity with the binding site of the ALDH2 protein. Specifically, ASIV can bind inside the pocket and form hydrogen bonds with GLU-340, ARG-329, TYR-456, and LYS-494 with docking energy less than or equal to −9.2 kcal/mol (Figure 8c), which indicates direct regulation of ALDH2. Altogether, these data suggest that ASIV might be a potential drug to alleviate the progression of renal fibrosis by directly and indirectly regulating ALDH2.

## 4. Discussion

In addition to the continuous deterioration of kidney function, CKD can also cause a series of complications, including renal hypertension, renal anemia, chronic kidney disease–mineral and bone abnormalities (CKD-MBD), etc. [52]. Currently, low-protein diet (LPD) interventions remain the primary recommendations in the conservative treatment to postpone the progression of CKD [53]. However, the application of LPDs in clinical practice still remains controversial due to the contraindications and side effects [54,55]. Thus, pharmacotherapy is crucial for treating CKD. Traditional Chinese medicine (TCM) has played a very large role in the prevention and treatment of CKD [56,57]. Recently, accumulating articles have reported that *Astragalus Mongholicus* (AM)*,* as a traditional Chinese herbal medicine, has a rich medicinal value, and recently, its anti-fibrosis effect has been increasingly investigated [58]. AS-IV, the main active component of saponins extracted from AM, has been reported to have various pharmacological activities, including regulation of the calcium ion equilibrium [59] and anti-oxidative [60], anti-apoptotic [61], and anti-fibrosis [62] functions. As an important stress-responsive system, autophagy has been proved to be involved in the pathogenesis of various kidney diseases, especially renal fibrosis [63,64]. Furthermore, autophagy is reportedly to be tightly involved in the regulation of EMT and the cell cycle [63,65]. In the present study, we found that ASIV could mitigate EMT and G2/M arrest by targeting and upregulating ALDH2 to inhibit autophagy in vivo and in vitro.

Autophagy is a dynamic process involving balancing intracellular energy and resources [66]. In addition to recycling organelles and proteins, autophagy can govern a range of cellular processes, including cell cycle, cell death, inflammation, metabolic pathways, and immune responses [67]. The role of autophagy in disease pathogenesis is complex, and it involves physiological or pathological regulation [68]. In contrast to the adaptive repair of autophagy after acute injury, the renal tubular epithelium is more prone to maladaptive outcomes after repeated and prolonged injury [69,70]. High levels of autophagy may lead to the over-digestion of cellular components, even including healthy organelles, resulting in cell death [22,23]. In addition, accompanied by irrevocable impairment in cells, sustained autophagy in the recovery phase inhibited the proliferation and regeneration of RTECs, which was detrimental to the kidney [71,72]. Additionally, it was reported that in a hyperuricemia nephropathy (HN) rat model, postponed treatment with 3-MA rescued tubular epithelial cells from G2/M arrest blunted EMT and suppressed tubulointerstitial fibroblasts activation [24]. Similarly, we found that ASIV could mitigate EMT and G2/M arrest by inhibiting autophagy in the CKD model. Previous studies have reported that the AKT/mTOR pathway is highly involved in the regulation of autophagy in kidney disease [73,74]. In the present study, we also demonstrated that ASIV inhibited autophagy by activating the AKT/mTOR signaling pathway. mTOR, as the downstream molecule of PI3K/AKT and a key intracellular sensor and mediator, plays a pivotal role in regulating autophagy [75]. Subsequently, we used rapamycin, an autophagy agonist that functions by inhibiting the phosphorylation of mTOR [76], to further investigate the role of mTOR-mediated autophagy in vitro. The results show that rapamycin indeed reversed the treatment effects of ASIV in TGF-β-induced EMT and G2/M arrest by promoting autophagy in HK-2 cells.

Previously, it was reported that the overexpression of ALDH2 alleviated excessive ECM deposition in primary idiopathic pulmonary fibrosis (IPF)-derived lung fibroblasts [77]. Contrarily, ALDH2-deficient mice were more susceptible to hepatic fibrosis and hepatocellular carcinoma under treatment with CCl4 plus alcohol [78]. Alda1, an ALDH2 agonist, was reported experimentally to ameliorate sympathetic excitation-induced cardiac fibrosis in mice [79] and mitigate necrosis and fibrosis in bile duct ligation mice [80]. Herein, we found that ASIV ameliorated EMT and G2/M arrest, as well as promoted the expression of ALDH2 in a concentration-dependent manner in vivo and in vitro, indicating that ALDH2 might be involved in the alleviation of renal fibrosis. In addition, it has been reported that ALDH2 activation improved cardiac diseases by inhibiting autophagy [81,82,83], and ALDH2 was also reported to regulate the AKT-mTOR signaling cascade to maintain cardiomyocyte survival homeostasis [84,85]. Then, it was reported that Alda-1 ameliorated tunicamycin-induced cardiomyocyte dysfunction by inhibiting autophagy, which was abolished by rapamycin [86]. Accordingly, in the present study, we found that ASIV increased the expression of ALDH2 and inhibited autophagy by activating the AKT/mTOR pathway in the mitigation of renal fibrosis, which was further validated by small RNA interference to knock down ALDH2 in TGF-β-induced HK-2 cells. Taken together, our data demonstrate that ASIV ameliorates renal fibrosis by promoting the expression of ALDH2 to regulate AKT/mTOR-mediated autophagy.

In the present study, we demonstrated that ASIV alleviates EMT and G2/M arrest in CKD models by increasing the expression of ALDH2 in vivo and in vitro. Meanwhile, the result of the dual-luciferase report assay indicates the activating effect of ASIV on the promoter of ALDH2. Furthermore, we used molecular docking to predicate and evaluate the potential combing sites between the ALDH2 protein and ASIV. However, the potential direct interaction warrants further validation and investigation. In addition, we adopted an adenine-induced CKD model, which has gained general acceptance as a classic model, as this intervention mimicked most of the structural and functional changes observed in progressive CKD in humans and did not require surgery or genetic manipulation [87]. However, there are many causes and complications of CKD, and the effects of ASIV on different CKD models should be further investigated in the future.

**Figure 9:**
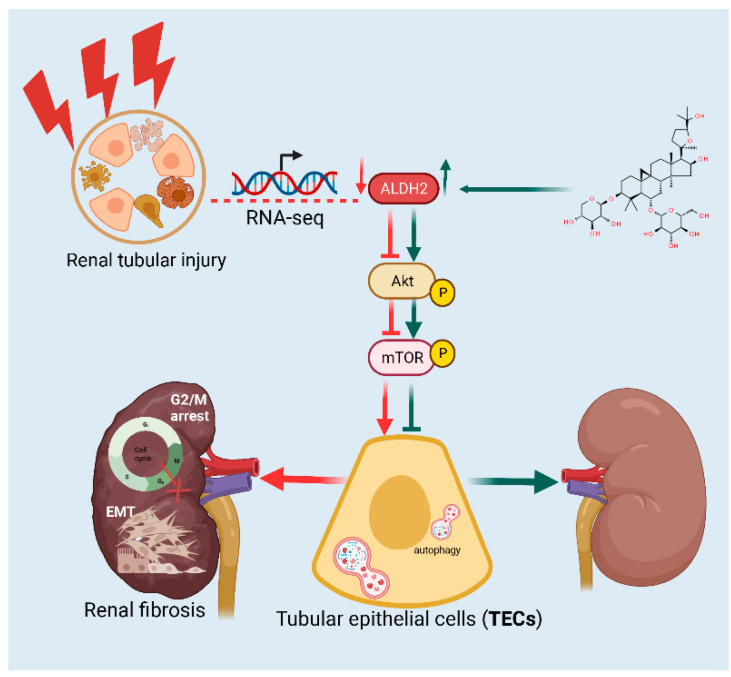
ASIV blunts EMT and G2/M arrest to alleviate renal fibrosis via regulating ALDH2-mediated autophagy.

In summary, the results of this study show that ASIV effectively treats renal fibrosis by alleviating EMT procession and G2/M arrest in vivo and in vitro, and this may be due to the ASIV-induced upregulation of the expression of ALDH2, which inhibits autophagy by regulating the AKT/mTOR pathway (Figure 9). Collectively, our data provide new evidence to further clarify the anti-fibrosis mechanism of ASIV in treating renal fibrosis. Thus, the present study indicates that ASIV is expected to become an effective drug for renal fibrosis and provides a valuable reference and guidance for clinical treatment.

## Data Availability

The data that support the findings of this study are available from the corresponding author on reasonable request.

## Supplementary Materials

The following supporting information can be downloaded at: https://www.mdpi.com/article/10.3390/cells12131777/s1, Table S1: Primers used in RT-qPCR Analysis. Figure S1: (a) Body weight, (b) serum Crea, and (c) serum BUN were measured when the modeling of rats was finished. *** *p* < 0.001 vs. the control group. Figure S2: Quantitative analysis of immunofluorescence for fibronectin, α-SMA, and p-H3/Ki67 in rat kidneys. Mean ± SEM. ### *p* < 0.001 vs. the control group; * *p* < 0.05, *** *p* < 0.001 vs. the adenine group. Figure S3: Gene set enrichment analysis (GSEA) for (a) extracellular matrix organization, (b) cell cycle, and (c) phagosome in kidney tissues. Figure S4: (a) Quantitative analysis of immunohistochemistry staining for ALDH2 in rat kidneys. (b) Quantitative analysis of immunofluorescence staining for LC3 in rat kidneys. Mean ± SEM. ### *p* < 0.001 vs. the control group; * *p* < 0.05, ** *p* < 0.01, *** *p* < 0.001 vs. the adenine group. Figure S5: (a) Cell viability was measured under treatment with TGF-β1 (10 ng/mL) or TGF-β1 (10 ng/mL) plus different concentrations of ASIV (10, 50, 100, and 150 μM). (b) Protein expression of LC3 I/II in HK-2 cells treated using Western blotting. (c) Quantitative analysis of the relative protein expression of LC3 II/LC3 I in the renal tissues. Mean ± SEM. *** *p* < 0.001. (d) Quantitative analysis of immunofluorescence for fibronectin, F-actin, p-H3/Ki67, and LC3 in TGF-β1-stimulated HK-2 cells. Mean ± SEM. ### *p* < 0.001 vs. the control group; *** *p* < 0.001 the TGF-β1 group. Figure S6: (a) Protein expression of ALDH2 in HK-2 cells treated with siNC or siALDH2 using Western blotting. (b) Quantitative analysis of the relative protein expression of ALDH2. (c) Quantitative analysis of immunofluorescence for fibronectin, F-actin, p-H3/Ki67, and LC3 in TGF-β1-stimulated HK-2 cells. Mean ± SEM. ### *p* < 0.001 vs. the control group; *** *p* < 0.001 vs. the TGF-β1 group; ^^^ *p* <0.001 vs. the TGF-β1 + ASIV group. Figure S7: (a) Protein expression of p-mTOR, t-mTOR, LC3 I/II, fibronectin, and p-H3ser10 in HK-2 cells using Western blotting. (b) Quantitative analysis of the phosphorylation level of mTOR and the relative protein expressions of p-mTOR, t-mTOR, LC3 I/II, fibronectin, and p-H3ser10. Mean ± SEM. ### *p* < 0.001 vs. the control group; * *p* < 0.05, ** *p* < 0.01, *** *p* < 0.001 vs. the rapamycin group. (c) Quantitative analysis of immunofluorescence for fibronectin, F-actin, p-H3/Ki67, and LC3 in TGF-β1-stimulated HK-2 cells. Mean ± SEM. ### *p* < 0.001 vs. the control group; ** *p* < 0.01, *** *p* < 0.001 vs. the TGF-β1 group; ^^ *p* < 0.01, ^^^ *p* < 0.001 vs. the TGF-β1 + ASIV group. Figure S8: Structure of ALDH2 promoter plasmid.
