# Supplementary material for: Astragaloside IV Blunts Epithelial–Mesenchymal Transition and G2/M Arrest to Alleviate Renal Fibrosis via Regulating ALDH2-Mediated Autophagy
Source: Cells. 2023 Jul 4;12(13):1777. doi: 10.3390/cells12131777 (PMC10340704; doi:10.3390/cells12131777)
Supplement: Supplementary file 1 [file cells-12-01777-s001.zip › cells-2414276-supplementary.pdf]

Supplementary Materials for

Astragaloside IV blunts epithelial-mesenchymal transition and G2/M arrest to alleviate renal fibrosis *via* regulating ALDH2-mediated autophagy

Table s1. Primers used in RT-qPCR Analysis.

Gene	Forward primer (5'→3')	Reverse primer (5'→3')
RN-Fn1	AAACCGGGAAGAGCAAGAG	CCGTTCCCACTGCTGATTTA
RN-Vim	CCATCAACACCGAGTTCAAGA	CGCACCTTGTCGATGTAGTT
RN-Acta2	AGGGAGTGATGGTTGGAATG	GGTGATGATGCCGTGTTCTA
RN-Tp53	ACATGACTGAGGTCGTGAGA	GATTTCCTTCCACCCGGATAAG
RN-Cdkn1a	AGTATGCCGTCGTCTGTTC	TCTCAGTGGCGAAGTCAAAG
RN-Aldh2	CTTCATTAACAATGAGTGGCATGA	CTTGTTCCCTTCGGCTACC
RN-Mtor	CAGACGCCAATGAGAGGAAG	AGGAGGTTCCGAAGGTAGTT
RN-Map1lc3b	CCACCAAGATCCCAGTGATTAT	TTCACGTGATCAGGTACAAGG
RN-Becn1	CAGGAACTCACAGCTCCATTAC	CCATCCTGGCGAGTTTCAATA
RN-Atg7	CACGAACTGACCCAGAAGAA	GGCAGAGTCACCATTGTAGTAA
RN-Gapdh	GACATGCCGCCTGGAGAAAC	AGCCCAGGATGCCCTTTAGT
HS-ALDH2	GGACATGGTCCTCAAATGTCTC	CGTGTGTAGCTGAAGAAGTCTC
HS-GAPDH	CAGGAGGCATTGCTGATGAT	GAAGGCTGGGGCTCATTT

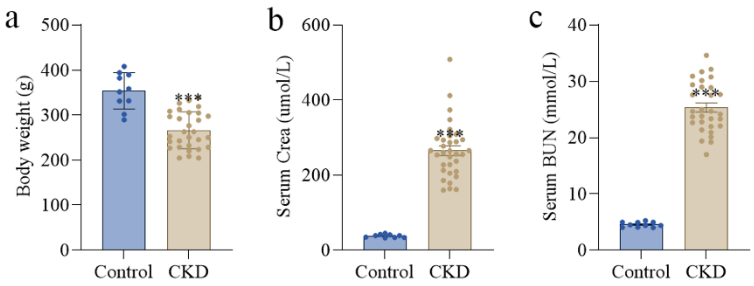

Figure s1. (a) Body weight, (b) Serum Crea and (c) Serum BUN were measured when the modeling of rats was finished. ***$p < 0.001$ vs the Control group.

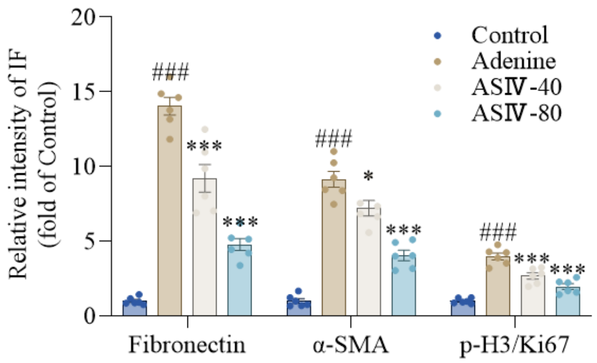

Figure s2. Quantitative analysis of immunofluorescence for Fibronectin, α-SMA and p-H3/Ki67 in rat kidneys. Mean ± SEM. ###$p < 0.001$ vs the Control group, *$p < 0.05$, ***$p < 0.001$ vs the Adenine group.

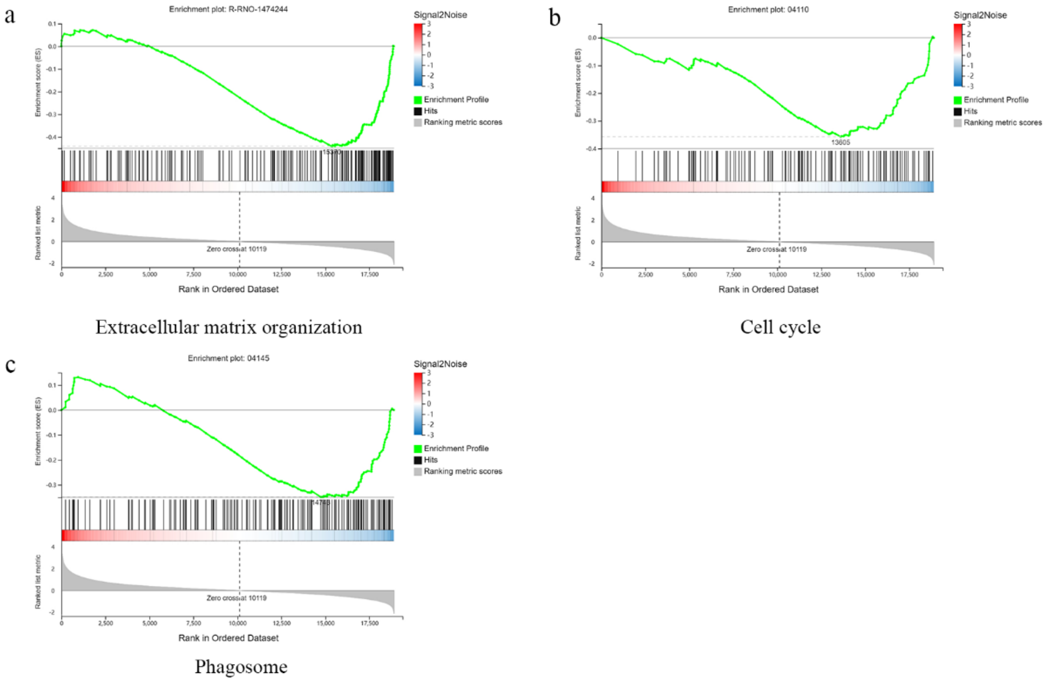

Figure s3. Gene Set Enrichment Analysis (GSEA) for (a) Extracellular matrix organization, (b) Cell cycle and (c) Phagosome in kidney tissues.

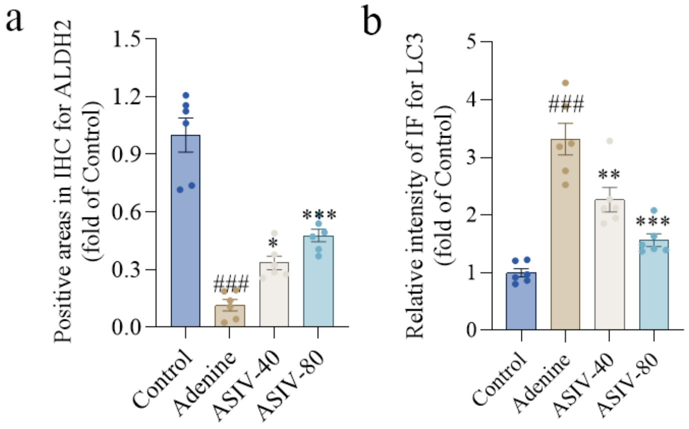

Figure s4. (a) Quantitative analysis of immunohistochemistry staining for ALDH2 in rat kidneys. (b) Quantitative analysis of immunofluorescence staining for LC3 in rat kidneys. Mean ± SEM. ###$p < 0.001$ vs the Control group, *$p < 0.05$, **$p < 0.01$, ***$p < 0.001$ vs the Adenine group.

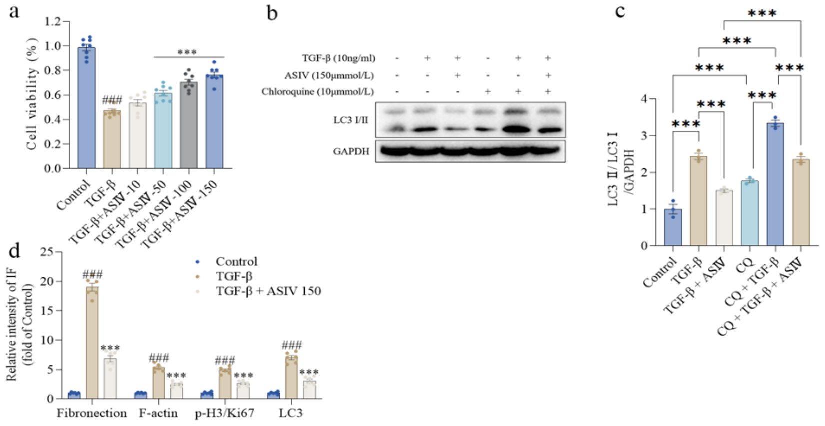

Figure s5. (a) Cell viability were measured under the treatment with TGF-β1 (10 ng/mL) or TGF-β1 (10 ng/mL) plus different concentrations of ASIV (10, 50, 100, 150 μM). (b) Protein expression of LC3 I/II in HK-2 cells treated by western blotting. (c) Quantitative analysis of the relative protein expression of LC3 II/LC3 I in the renal tissues. Mean ± SEM. ***$p < 0.001$. (d) Quantitative analysis of immunofluorescence for Fibronectin, F-actin, p-H3/Ki67 and LC3 in TGF-β1-stimulated HK-2 cells. Mean ± SEM. ###$p < 0.001$ vs the Control group, ***$p < 0.001$ the TGF-β1 group.

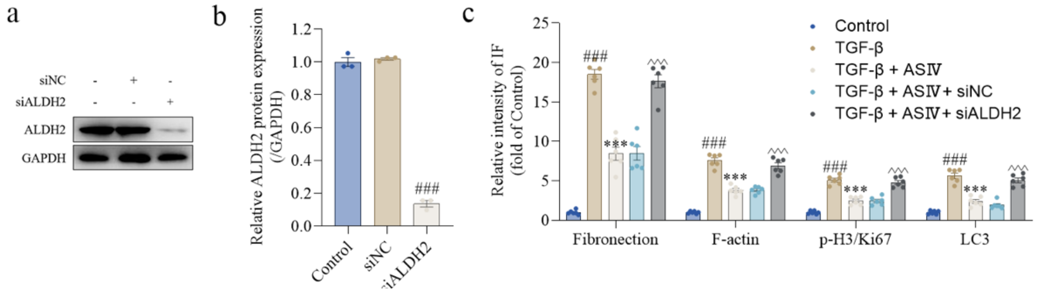

Figure s6. (a) Protein expression of ALDH2 in HK-2 cells treated with siNC or siALDH2 by western blotting. (b) Quantitative analysis of the relative protein expression of ALDH2. (c) Quantitative analysis of immunofluorescence for Fibronectin, F-actin, p-H3/Ki67 and LC3 in TGF-β1-stimulated HK-2 cells. Mean ± SEM. ###$p < 0.001$ vs the Control group, ***$p < 0.001$ the TGF-β1 group, ^^^p <0.001 vs the TGF-β1+ASIV group.

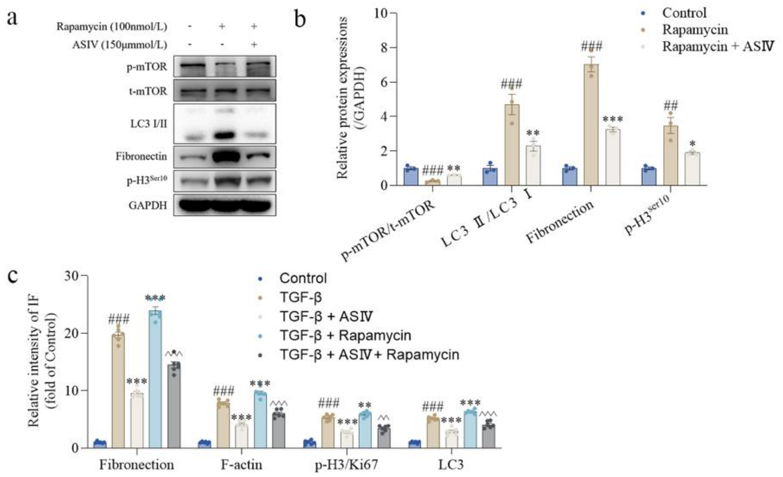

Figure s7. (a) Protein expression of p-mTOR, t-mTOR, LC3 I/II, Fibronectin and p-H3^{ser10} in HK-2 cells treated by western blotting. (b) Quantitative analysis of the phosphorylation level of mTOR and the relative protein expressions of p-mTOR, t-mTOR, LC3 I/II, Fibronectin and p-H3^{ser10}. Mean ± SEM. ###$p < 0.001$ vs the Control group, *$p < 0.05$, **$p < 0.01$, ***$p < 0.001$ vs the Rapamycin group. (c) Quantitative analysis of immunofluorescence for Fibronectin, F-actin, p-H3/Ki67 and LC3 in TGF-β1-stimulated HK-2 cells. Mean ± SEM. ###$p < 0.001$ vs the Control group, **$p < 0.01$, ***$p < 0.001$ vs the TGF-β1 group, ^^p <0.01, ^^^p <0.001 vs the TGF-β1+ASIV group.

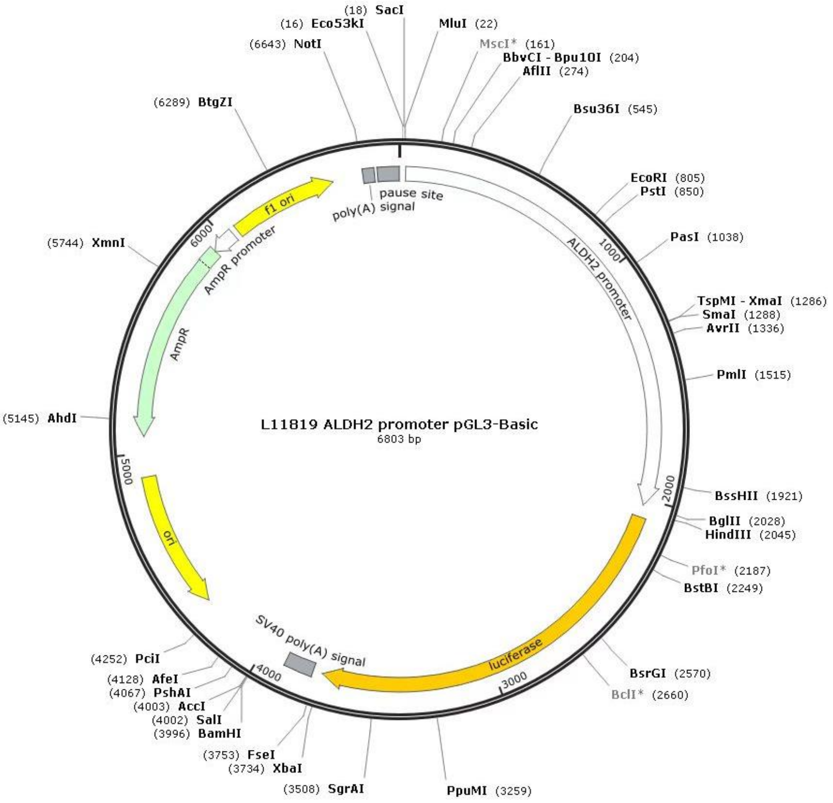

Figure s8. Structure of ALDH2 promoter plasmid.
